# Spatial and temporal analysis of China’s healthcare resource allocation measurements based on provincial data: 2010–2021

**DOI:** 10.3389/fpubh.2023.1269886

**Published:** 2023-11-21

**Authors:** Hengna Ren, Chen Li, Yi Huang

**Affiliations:** ^1^Business School, University of Shanghai for Science and Technology, Shanghai, China; ^2^School of Management, Shanghai University of Engineering Science, Shanghai, China; ^3^School of Geographic Science, Nantong University, Nantong, China

**Keywords:** healthcare resource allocation, regional differences, spatial–temporal pattern, spatial autocorrelation, China provinces

## Abstract

**Background:**

With the development of society, industrialization, urbanization, aging, lifestyle and social transformation, environmental degradation, global warming and other factors have had a great impact on the health of the population, and there is an urgent need to take a series of practical actions to promote the improvement of national health. Among them, healthcare resource allocation plays a key role in advancing the level of national health, treatment of chronic diseases, and leisure and healthcare.

**Methods:**

This article collected panel data on healthcare resource allocation in all provinces of China from 2010 to 2021, and comprehensively applied Analytic Hierarchy Process, comprehensive scoring method, regional difference analysis and spatial autocorrelation analysis to reveal regional differences, spatial–temporal patterns and development characteristics of healthcare resource allocation in China.

**Results:**

In terms of regional differences, intra-regional differences in healthcare resource allocation tend to narrow and inter-regional differences tend to widen. In terms of spatial pattern, the western provinces on the left side of the Hu Huanyong line generally have higher scores, while the central and eastern provinces on the right side of the Hu Huanyong line have lower scores, and healthcare resource allocation in the provinces on the left side of the Hu Huanyong line, such as Tibet, Xinjiang, Qinghai, Ningxia, Gansu, Inner Mongolia, Sichuan, have the spatial characteristics of HH clusters in terms of geographic location, while the southeast coastal provinces, such as Zhejiang, Fujian, Guangdong, Hainan, have the spatial characteristics of LL clusters in terms of geographic location. From the quadrant analysis, the 2010–2021 healthcare resource allocation in the first quadrant concentrates most of the provinces in the western and northeastern regions, while the third quadrant concentrates most of the provinces in the eastern region.

**Conclusion:**

The allocation of healthcare resources in China’s four major zones has undergone a process of change from “unbalanced quantity to relatively balanced quantity,” but high-quality healthcare resources are highly concentrated in the eastern part of the country, and the problem of contradiction between people and doctors is prominent. It is recommended that Internet plus healthcare technology be used to reshape the regional allocation of high-quality healthcare resources.

## 1 Introduction

The optimal allocation of healthcare resources is an important guarantee for achieving sustainable economic and social development, and is the key to enjoying basic healthcare services for all, meeting people’s health needs, and promoting the construction of a healthy China (1). The level of healthcare resource allocation in a country or region is not only directly related to people’s health, but also affects the smooth development of the economy and society (2). In the process of China’s economic development from high-speed growth to high-quality development, the contradiction between the people’s growing health needs and the imbalance and insufficiency of healthcare resource allocation remains prominent. Reasonable resource allocation can better meet the healthcare service needs of different groups of people, improve people’s quality of life and health, reduce the gap in healthcare resource allocation between regions, between urban and rural areas, and between the rich and the poor, and realize the equity of healthcare resource allocation, which will in turn improve the stability and sustainability of the healthcare resource allocation system. From the perspective of geographic space, healthcare spatial distribution profoundly affects the health level and quality of life of residents in different regions, and is a problem of resource spatial allocation. Currently, academics mainly discuss healthcare resource allocation from two aspects.

First, healthcare resource allocation equity and efficiency research. Equity and efficiency have been important issues in healthcare resource allocation (3), and there is a value-oriented game (4). Academics have discussed the issue of healthcare resource equity and efficiency from several aspects of spatial equity, differential allocation, and measurement methods. First, spatial equity. The efficiency of resource allocation should take into account the interests of different groups and spatially balance efficiency and equity. Spatial equity-oriented healthcare resource allocation optimization can be achieved through the maximum accessibility parity model (5), and researchers have conducted healthcare resource allocation equity and efficiency studies from a spatial perspective in China (6, 7), the United States (8), Russia (9), and Thailand (10), respectively. The second is differential allocation. The prerequisite for effective healthcare resource allocation is differential allocation, so as to achieve equal rights, equal opportunities, procedural equality and fairness of results (11). Effective healthcare resource allocation is oriented to regional demand, providing differentiated supply and strengthening inter-regional exchanges so as to maximize efficiency (12). Third, the measurement method. Scholars use the Gini coefficient and the DEA-Malmquist index to comprehensively measure and characterize the efficiency and equity of spatial allocation of healthcare resources in China’s provincial healthcare resource allocation, the county-level healthcare resource allocation in Hubei province (13), and the Xinjiang region (14), respectively, and explore their influencing factors. Using the concentration index, Theil index and comprehensive evaluation method, we analyze the equity of healthcare resource allocation at the grassroots level (15), the balance of spatial allocation of healthcare resources at each level and the changing status of healthcare resources in Shanghai, China, from 2012 to 2021 (16). Radial super-efficiency model and Kernel density estimation were used to analyze the trajectory of healthcare allocation efficiency changes (17) and dynamic evolution (18) in China.

Second, the study of regional differences in healthcare resource allocation and spatial distribution. The researchers studied regional differences in healthcare resource allocation from three spatial scales: the urban cluster perspective, the provincial perspective, and the municipal perspective. The first is the provincial scale. China’s provincial healthcare resource allocation shows a rapid development trend. Quantitatively, the number of healthcare resources in China has increased, but the overall effective allocation has not been realized (19). In terms of regional differences, there are differences in the efficiency of healthcare resource allocation in different provinces, with the highest efficiency in the eastern region compared to the western region, followed by the central region. Healthcare resource allocation is uneven, but has shown a fluctuating and narrowing trend in recent years (20). In terms of measurement indicators, the number of primary healthcare institutions, the number of beds in primary healthcare institutions, and the number of primary healthcare personnel (21) are generally used to analyze the three dimensions. The second is city cluster scale. Healthcare resource allocation ownership decreases with decreasing city cluster level, and the difference in healthcare resource allocation increases with decreasing city cluster level (22). The spatial correlation of healthcare resource allocation in urban agglomerations and its convergence characteristics were measured by three dimensions of healthcare resource allocation supply, demand and efficiency (23). The study shows that the quality resources in the Yangtze River Delta, Beijing-Tianjin-Hebei, and the middle reaches of the Yangtze River urban agglomerations are higher than their average levels. The third is city scale. Scholars have analyzed the spatial pattern of healthcare resource allocation, influencing factors and its network evolution characteristics in Beijing (24), Wuhan (25), Zhengzhou (26), and Chongqing (27), respectively. A comparative study of the spatial distribution of healthcare resources between Beijing and London, Paris, New York and New Delhi is conducted through the dimensions of “proportion of healthcare land area in urban area” and “*per capita* area of healthcare land,” and it is found that the center of the region has a high concentration of healthcare resources, which is the most important factor for the development of healthcare resources. The study found that the concentration of healthcare resources in urban areas is obvious, and the spatial distribution of resource allocation varies greatly, so the optimization of spatial distribution can be achieved by strengthening the management of control regulations, innovating healthcare models, coordinating the development of the region (28), and reinforcing the power of grassroots healthcare (29, 30).

In summary, the above studies provide an open idea for healthcare resource allocation measurement, but there are some areas that deserve further exploration. Healthcare resource allocation involves the dual considerations of equity and efficiency, but the two are often in conflict. As healthcare resources are always limited (doctors, hospitals, drugs, etc.), their benefits to different populations need to be weighed when resources are allocated. Different populations and regions have different needs for healthcare resource allocation, which also leads to the possibility of sacrificing efficiency while pursuing equity. In a remote rural area and a large city, the same investment in healthcare resource allocation may result in different health outcomes. Rural areas may require more resources to achieve the same health outcomes as urban areas due to poor infrastructure and low levels of education. Studies have been conducted to strengthen the measurement of equity and efficiency of healthcare resource allocation and to analyze healthcare resource allocation from the perspective of regional differences. In recent years, China has increased the investment and spatial allocation of healthcare resources in each region, but existing studies have not sufficiently analyzed the developmental characteristics of healthcare resource allocation in different regions, and have not sufficiently explored the regionality and regularity of healthcare resource allocation at the national level. Based on this, we will construct the panel data of healthcare resource allocation by province from 2010 to 2021, and comprehensively use the AHP method, comprehensive scoring method, Theil index, and Moran’s I index to measure the regional differences, spatial–temporal characteristics, and spatial–temporal pattern of healthcare resource allocation in each province, in order to promote the optimal allocation of healthcare resources in China. Healthcare resource allocation, and provide empirical evidence for promoting the optimal allocation of healthcare resources in China.

## 2 Data and methods

### 2.1 Research methods

#### 2.1.1 Analytic hierarchy process

In 1970, Saaty proposed Analytic Hierarchy Process (AHP), which is a systematic and hierarchical method of analysis that combines qualitative and quantitative. AHP method divides the evaluation objectives into an objective level (A), a criterion level (B), and a program level (C). By comparing two by two, the weight of each criterion on the objectives *w_i_* be determined, which is characterized by subjective assignment.

AHP usually uses a 1–9 scale to judge the relative importance of each indicator in the system being evaluated, thus creating a judgment matrix. Let the evaluation element be X = {*x_1_*,*x_2_*,...,*x_i_*,..., *x_n_*}, *x_ij_* denotes the result of the comparison of the importance of xi relative to *x_j_*, and the 1–9 scale is used. Based on the meaning of the *x_ij_* value scale, the Delphi method, that’s taking the average scoring of experts, is used to compare the elements of the assessment element set X two by two, so as to obtain the judgment matrix P. The judgment matrix satisfies (31):


(1) $$P=X_{n\times n}$$

The judgment matrix X_n × n_ has *x_ij_* = 1/*x_ji_*. The eigenvector M_n × 1_ corresponding to the maximum eigenvalue *λ_max_* is measured using the equation PM = *λ_max_*, and the weights *w_i_* of each evaluation index are obtained using the normalized eigenvector M, which can be measured using the sum-product method.

In order to judge the scientific of the obtained weights, it is necessary to introduce the consistency indicator *CI* to test the consistency of the judgment matrix.


(2) $$CI=\frac{\lambda_{\text{max}}-n}{n-1}$$

The greater the value of consistency *CI*, the greater the degree of deviation of the judgment matrix from full consistency, the smaller the value of *CI*, the better the consistency of the matrix, when *CI* takes the value of 0, it indicates that the matrix has full consistency.

In order to test whether the judgment matrix has satisfactory consistency, it is necessary to define the test coefficient *CR*.


(3) $$CR=\frac{CI}{RI}$$

If *CR* < 0.1, it means that the judgment matrix passes the consistency test, and vice versa, the judgment matrix needs to be adjusted until it passes the consistency test, where *RI* (Random Index) is the Random Consistency Indicator, and the *RI* can be obtained from the average Random Consistency Indicator lookup table.

#### 2.1.2 Composite score method

The study used the composite score method to measure healthcare resource allocation in each province of China. The composite score of healthcare resource allocation was composed of Healthcare Facilities, Healthcare Personnel, and Healthcare Beds secondary indicators, and the secondary indicators were composed of nine tertiary indicators. Due to the differences in the magnitude of the raw data, the indicators were processed with data dimensionless by drawing on existing studies (32). The formula for measuring the composite score is as follows:


(4) $$\left\{ \begin{array}{l} d_{ij}=\frac{x_{ij}-x_{ij\min}}{x_{ij\max}-x_{ij\min}}\left(\mathrm{Positive}\;\mathrm{Indicator}\right)\\ d_{ij}=\frac{x_{ij\max}-x_{ij}}{x_{ij\max}-x_{ij\min}}\left(\mathrm{Negative}\;\mathrm{Indicator}\right) \end{array}\right.$$

(5) $$\begin{array}{l} Z=Z_{HF}+Z_{HP}+Z_{HB}\\ \overset{h}{\underset{n=1}{\sum }}w_{i}d_{ij}=\overset{h_{HF}}{\underset{n=1}{\sum }}w_{i}d_{HF_{ij}}+\overset{h_{HP}}{\underset{n=1}{\sum }}w_{i}d_{HP_{ij}}+\overset{h_{HB}}{\underset{n=1}{\sum }}w_{i}d_{HB_{ij}} \end{array}$$

In the above equation, *Z* is the comprehensive score of healthcare resource allocation, *Z_HF_*, *Z_HP_* and *Z_HB_* are the scores of secondary indicators of Healthcare Facilities, Healthcare Personnel and Healthcare Beds, respectively, and *x_ij_* denotes the raw data of healthcare resource allocation indicators. *x*_*ij*min_ and *x*_*ij*max_ denote the minimum and maximum values of the original data, respectively. Dij denotes the composite score of healthcare resource allocation after dimensionless processing, and *w_i_* is the weight measured by AHP method.

#### 2.1.3 Theil index

The Theil index examines inequality and variability from the concepts of informativeness and entropy, it decomposes the overall variability into variability between parts and variability within parts, and has a wide range of applications for analyzing and decomposing variability. The composite entropy index examines the variability between individuals from the concepts of informativeness and entropy, and it is the expected value of informativeness, i.e., the amount of expected information. The closer the relationship between individuals, the smaller the combined entropy index (33, 34).


(6) $$GE=\{\begin{array}{l} \overset{n}{\underset{i=1}{\sum }}p_{i}\left[{\left(y_{i}/u\right)}^{c}:1\right],c\neq 0,1\\ \overset{n}{\underset{i=1}{\sum }}p_{i}\left(y_{i}/u\right)\mathrm{lg}\left(y_{i}/u\right),c=1\\ \overset{n}{\underset{i=1}{\sum }}p_{i}\mathrm{lg}\left(y_{i}/u\right),c=0 \end{array}$$

In the above equation, the parameter c is used to determine the sensitivity of the index change. In general, it determines the sensitivity of the exponential change when c < 2. When c = 0, 1, it is well known as Theil index.

Due to its property of dividing the overall variation into within-zone and between-zone variation, Theil index is widely used in empirical studies of overall spatial heterogeneity as well as spatial heterogeneity, and is calculated by the formula:


(7) $$\mathrm{Theil}=\overset{n}{\underset{i=1}{\sum }}T_{i}\ln\left({nT}_{i}\right)=T_{WR}+T_{BR}$$

The Theil index can be further decomposed into intra-zonal and inter-zonal differences if the area under study is divided into groups according to a certain methodology.


(8) $$\begin{array}{l} T_{WR}=\overset{n_{db}}{\underset{i=1}{\sum }}T_{i}\ln\left(n_{db}\frac{T_{i}}{T_{db}}\right)+\overset{n_{d}}{\underset{i=1}{\sum }}T_{i}\ln\left(n_{d}\frac{T_{i}}{T_{d}}\right)+\\ \overset{n_{z}}{\underset{i=1}{\sum }}T_{i}\ln\left(n_{z}\frac{T_{i}}{T_{z}}\right)+\overset{n_{x}}{\underset{i=1}{\sum }}T_{i}\ln\left(n_{x}\frac{T_{i}}{T_{x}}\right) \end{array}$$

(9) $$\begin{array}{l} T_{BR}=T_{db}\ln\left(T_{db}\frac{n}{n_{db}}\right)+T_{d}\ln\left(T_{d}\frac{n}{n_{d}}\right)+\\ T_{z}\ln\left(T_{z}\frac{n}{n_{z}}\right)+T_{x}\ln\left(T_{x}\frac{n}{n_{x}}\right) \end{array}$$

In the above equations, Theil denotes Theil index; n is the number of provincial units within the sample region; T_WR_ is the difference within the four regional zones of the Northeast, East, Central, and West; T_BR_ is the difference between the four regional zones. n_db_, n_d_, n_z_, and n_x_ are the number of provincial units within the Northeast, East, Central, and West regions, respectively; T_i_ is the ratio of the measured indicators within the region to the national average ratio T_db_, T_d_, T_z_, T_x_ are the ratio of measured indicators to the national average in the Northeast, East, Central, and West regions, respectively.

The study covers 31 provinces, municipalities directly under the central government, autonomous regions and other provincial administrative units (Hong Kong SAR, Macao SAR and Taiwan Province are not included in the evaluation for the time being due to missing data). The four major zones measured in the article are: the northeastern region, which includes 3 provincial administrative units, namely Liaoning, Jilin and Heilongjiang; and the eastern region, which includes 10 provincial administrative units, namely Beijing, Tianjin, Hebei, Shanghai, Jiangsu, Zhejiang, Shandong, Fujian, Guangdong and Hainan. The central region includes 6 provincial administrative units, including Shanxi, Henan, Anhui, Hubei, Hunan and Jiangxi; the western region includes 12 provincial administrative units, including Inner Mongolia, Chongqing, Sichuan, Guangxi, Guizhou, Yunnan, Shaanxi, Gansu, Ningxia, Tibet, Qinghai and Xinjiang.

#### 2.1.4 Spatial autocorrelation analysis

Spatial autocorrelation is the degree of correlation between a certain geographic phenomenon or attribute value on a regional unit and the same phenomenon or attribute value on a neighboring geographic unit (35), and is divided into global spatial autocorrelation and local spatial autocorrelation. Global spatial autocorrelation is mainly measured by Moran’s I index, and its calculation formula is as follows:


(10) $$I=\left(\frac{n}{\underset{i}{\sum }\underset{j}{\sum }W_{ij}}\right)\left(\frac{\underset{i}{\sum }\underset{j}{\sum }W_{ij}\left(x_{i}-\bar{x}\right)\left(y_{i}-\bar{y}\right)}{\underset{i}{\sum }{\left(x_{i}-\bar{x}\right)}^{2}}\right)$$

Where: *W_ij_* is the spatial matrix; n is the number of regional cells, *x_i_* is the observation of the *i^th^* cell; and $\bar{x}$ is the mean value of the observation. The expected value of Moran’s I index is:


(11) $$E\left(I\right)=-1/\left(n-1\right)$$

Under the premise of passing the test of significance, a positive Moran’s I index indicates that regions with higher composite scores of healthcare resource allocation show significant spatial clustering; a positive Moran’s I index indicates that regions with their neighboring regions have significant spatial differences in their composite scores of healthcare resource allocation. Scores have significant spatial differences.

Local spatial autocorrelation is used to reveal the heterogeneous characteristics of geospatial differences to comprehensively reflect the trend of regional differences in the composite score of healthcare resource allocation in each province, and is usually measured by the Local Moran’s I index. The degree of spatial difference between an attribute value and its neighboring regions and the significance of the difference. For spatial unit *i*, its Local Moran’s I is defined as:


(12) $$I_{i}=z_{i}\underset{i}{\sum }W_{ij}z_{j}$$

Where: *z_i_* and *z_j_* are standardized values of observations on district *i* and district *j*; *W_ij_* is the spatial weight. The local spatial autocorrelation reflects the clustering characteristics of the district healthcare resourcing composite score through the LISA clustering map.

### 2.2 Evaluation indicators and weights

The AHP method was used to measure the weights of secondary and tertiary indicators (Table 1).

**Table 1:** Weights established by analytic hierarchical analysis.

Secondary indicators	Weights of secondary indicators	Tertiary indicators	Weights of tertiary indicators
Healthcare facilities	0.3324	Number of hospitals per 10,000 people	0.1100
		Number of primary health-care institutions per 10,000 persons	0.1145
		Number of specialized public health institutions per 10,000 people	0.1079
Healthcare Personnel	0.3596	Ratio of health technicians to urban/rural	0.1114
		Number of practicing (assistant) physicians per 10,000 persons	0.1284
		Registered nurses per 10,000 population	0.1198
Healthcare Beds	0.3080	Number of hospital beds per 10,000 people	0.1050
		Number of beds in primary health-care institutions for 10,000 persons	0.0993
		Number of beds in specialized public health institutions per 10,000 people	0.1037

Firstly, the expert scoring of the secondary and tertiary indicators is done through the Delphi method. Secondly, the average score was obtained and the importance of the indicators was ranked to establish a judgment matrix. Thirdly, the sum and product method is used to measure the weights and verify the consistency of the judgment matrix.

The judgment matrix for the secondary indicators was constructed as follows:


$$B=\left|\begin{array}{ccc} 1 & 0.9500 & 1.0500\\ 1.0526 & 1 & 1.2000\\ 0.9524 & 0.8333 & 1 \end{array}\right|$$

Derive the judgment matrix weights:


$$w=\left|0.3324\;0.3596\;0.3080\right|$$

Consistency tests were performed on the judgment matrix:


$$\lambda_{\text{max}}=3.000752$$

$$CI=\frac{\lambda_{\text{max}}-n}{n-1}=\frac{3.000752-3}{3-1}=0.000376$$

$$CR=\frac{CI}{RI}=\frac{0.00376}{0.58}=0.000648<0.1$$

The judgment matrix passes the consistency test.

Similarly, the judgment matrices of the three levels of indicators were constructed separately, the judgment matrix weights were derived, and the consistency test was conducted.

### 2.3 Data sources

We fully consider the accessibility and continuity of the data sources of healthcare resource allocation by province, and all the nine three-level indicators of healthcare resource allocation are derived from the China Statistical Yearbook 2001–2022 compiled by the National Bureau of Statistics and published by China Statistical Publishing House, which corresponds to the panel data of the indicators of medical care, doctors, and beds of 31 provincial administrative units nationwide in the period of 2000–2021.

## 3 Results and analyzes

### 3.1 Analysis of regional differences in healthcare resource allocation

**Figure 1:**
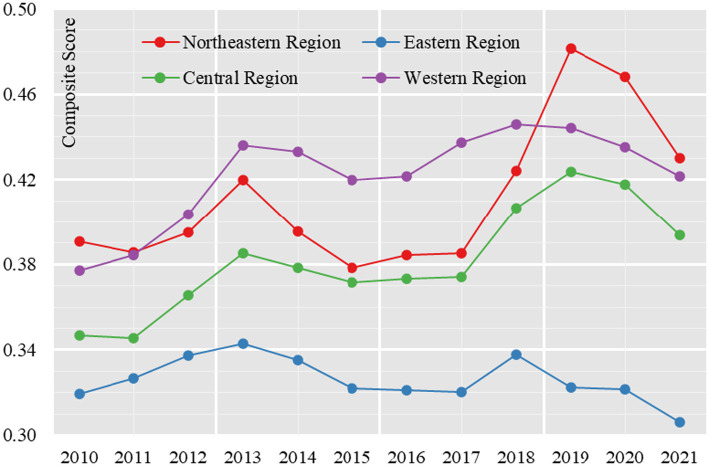
Healthcare resource allocation composite score of China (2010–2021).

From 2010 to 2021, the composite score of healthcare resource allocation in the four major zones generally shows a fluctuating trend of growth, which is reflected in the composite score going through the process of “rising-declining-rising-declining.” The average score of the four major zones increased from 0.3586 in 2010 to 0.3961 in 2013, then decreased to 0.3729 in 2015, then increased to 0.4179 in 2019, and then decreased to 0.3879 in 2021, with an average score increase of 8.17 percentage points from 2010 to 2021, indicating that healthcare resource allocation in the four major zones has increased to some extent. During the study period, the comprehensive score of healthcare resource allocation in the east, middle and west has obvious gradient characteristics. 2010–2018, the western region’s comprehensive score of healthcare resource allocation was ahead of the other three regions, showing a spatial and temporal pattern of “western region > northeastern region > central region > eastern region.” From 2019 to 2021, the Northeast region was ahead of the other three regions, showing a spatial and temporal pattern of “Northeast region > Western region > Central region > Eastern region” (Figure 1).

In terms of regional variation coefficients, the total variation in the composite score of healthcare resource allocation shows a narrowing trend, in which the intra-zone variation tends to narrow and the inter-zone variation tends to widen (Table 2). Theil index consists of the total variation, intra-zone variation, and inter-zone variation, which is equal to the sum of intra-zone and inter-zone variation, and is divided into the northeastern intra-zone, the eastern intra-zone, the central intra-zone, and the western intra-zone variations. The total variation was equal to the sum of intra- and inter-zonal variation, and intra-zonal variation was divided into northeastern intra-zonal variation, eastern intra-zonal variation, central intra-zonal variation, and western intra-zonal variation. The Theil index of the total difference in the composite score decreases from 0.0326 in 2010 to 0.0263 in 2021, a decrease of 19.45%. In terms of intra-zone differences, the Theil index decreases from 0.0296 in 2010 to 0.0165 in 2021, with a decrease of 44.20%, of which the changes in the Theil index of the differences within the Northeast region, the differences within the East region, the differences within the Central region, and the differences within the West region are −0.55, −17.48%, 75.81%, and 63.46 per cent. In terms of inter-zone variation, the Theil index increases from 0.0030 in 2010 to 0.0097 in 2021, a 2.29-fold increase.

**Table 2:** Theil index decomposition of healthcare resources allocation in China (2010–2021).

Year	T_db_	T_d_	T_z_	T_x_	T_WR_	T_BR_	T
2010	0.0002	0.0080	0.0075	0.0139	0.0296	0.0030	0.0326
2011	0.0003	0.0070	0.0049	0.0124	0.0246	0.0027	0.0273
2012	0.0003	0.0056	0.0039	0.0091	0.0189	0.0030	0.0218
2013	0.0002	0.0070	0.0032	0.0072	0.0176	0.0052	0.0228
2014	0.0006	0.0065	0.0043	0.0056	0.0170	0.0057	0.0227
2015	0.0005	0.0064	0.0055	0.0064	0.0187	0.0060	0.0248
2016	0.0003	0.0069	0.0060	0.0049	0.0181	0.0064	0.0245
2017	0.0010	0.0077	0.0051	0.0045	0.0183	0.0084	0.0267
2018	0.0003	0.0091	0.0055	0.0025	0.0173	0.0067	0.0241
2019	0.0004	0.0071	0.0048	0.0029	0.0152	0.0108	0.0261
2020	0.0001	0.0074	0.0025	0.0017	0.0118	0.0096	0.0214
2021	0.0002	0.0094	0.0018	0.0051	0.0165	0.0097	0.0263

**Figure 2:**
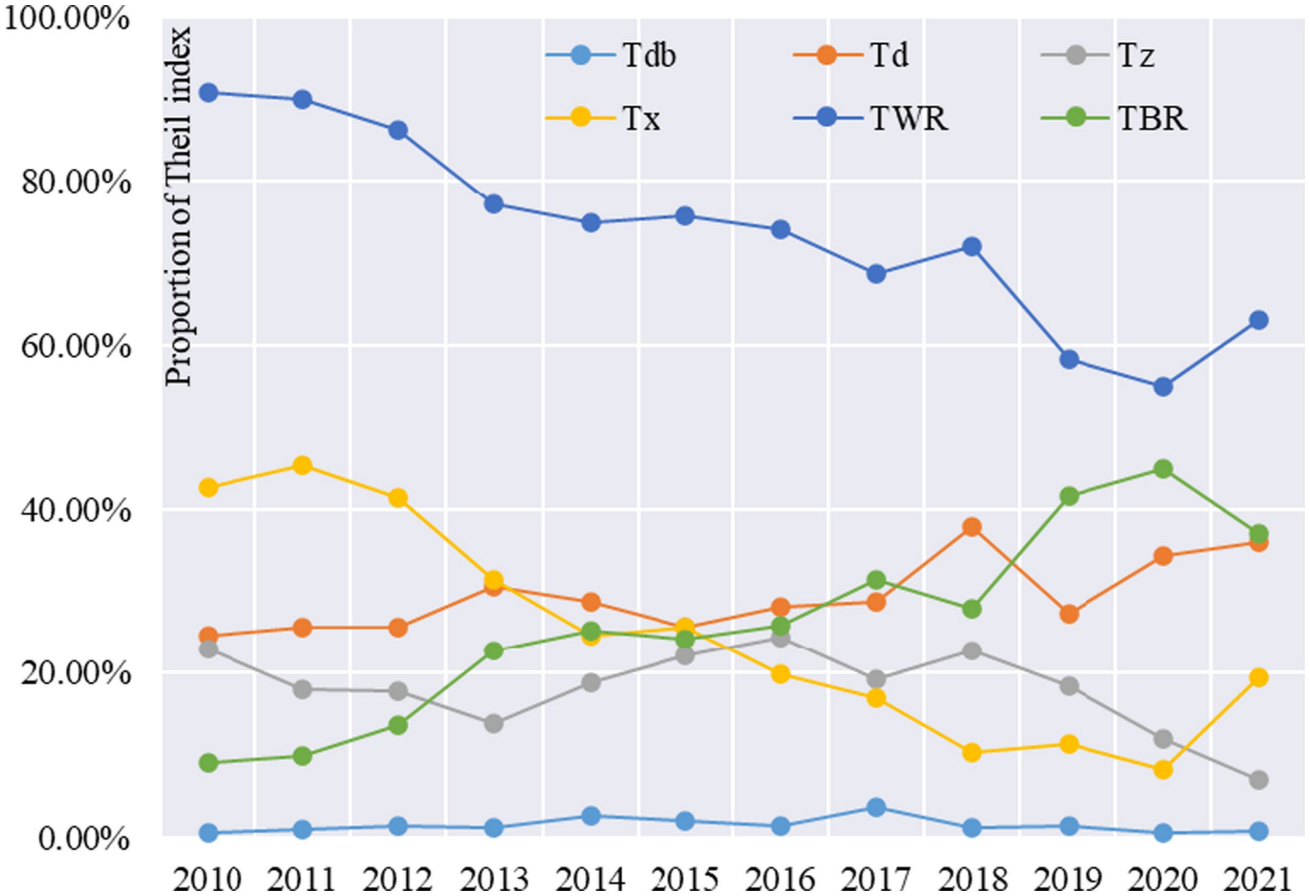
Contribution of Theil index to healthcare resource allocation in China (2010–2021).

In terms of the contribution rate of regional difference coefficients, the contribution rate of intra-zone differences shows a decreasing trend during the study period, from 90.93 per cent in 2010 to 62.99 per cent in 2021 (Figure 2). The contribution rate of inter-zone differences shows an increasing trend, from 9.07% in 2010 to 37.01% in 2021, which indicates that the differences between the four major zones tend to expand, while the differences within the zones tend to decrease. The contribution rates of intra-zone differences among the four major regions, in descending order, are: Eastern region > Western region > Central region > Northeastern region. The contribution rate of intra-zone differences in the eastern region rises rapidly from 24.66% in 2010 to 35.96% in 2021, indicating that healthcare resource allocation tends to cluster in the eastern region; the contribution rate of intra-zone differences in the western region falls rapidly from 42.73% in 2010 to 19.38% in 2021, indicating that the western region tends to flatten the change in healthcare resource allocation. The contribution rate of difference within the western region drops rapidly from 42.73% in 2010 to 19.38% in 2021, indicating a clear trend of flattening of resource allocation in the western region. The contribution rate of intra-zone variance in the Central Region also shows a rapid decline, with the contribution rate of intra-zone variance in the Central Region rapidly declining from 22.94% in 2010 to 6.89% in 2021, indicating that the trend of flattening the allocation of healthcare resources in the Central Region is also obvious. The Northeast region has the lowest intra-zone variance contribution (only 0.76 per cent) and the variation over the study period is very small.

Characterizations of changes in regional differences in healthcare allocation:

First, the trend of regional healthcare resource allocation and inputs increasing at different speeds is obvious. Since 2012, the Chinese government has continuously improved the new mechanism for the operation of grassroots healthcare organizations, pushed forward the reform of public hospitals and a series of other efforts, greatly promoting the development of the healthcare cause, and putting forward the institutional framework of healthcare with Chinese characteristics in the form of a public healthcare service system, a healthcare service system, a healthcare guarantee system, and a drug supply and guarantee system. The institutional framework of healthcare with Chinese characteristics, and promoting the process of equalizing the spatial allocation of healthcare resources, thereby significantly increasing the number of hospitals in the northeast, east, central and west regions from 2,306, 7,303, 4,982 and 6,327, respectively, in 2010 to 3,456, 12,808 and 9,042, respectively, 11,309, an increase of 1,150, 5,505, 4,060 and 4,982, respectively.

Second, the number of regional healthcare resource allocation tends to be balanced. The rise in the overall score of healthcare resource allocation in the western region has narrowed the difference in the quantity of healthcare resource allocation between the eastern and western regions, indicating that during the period under study, the allocation of healthcare resources in China’s four major zones went through the process of “unbalanced quantity - relatively balanced quantity,” which reflects that the level of equalization of public services in healthcare resources in the four major zones is constantly taking a new step forward.

Third, there are obvious differences in the quality of regional healthcare resource allocation. Due to historical reasons, differences in the level of economic development and other reasons, regional differences in the allocation of high-quality healthcare resources in the east, center and west still exist and are large, with the eastern region having 1,810,879 practicing (assistant) doctors in 2021, which is 5.73, 1.71 and 1.64 times that of the northeastern, central and western regions, respectively. In this regard, in 2020, the Chinese government issued the Opinions on Deepening the Reform of the Medical Security System, which aims to provide opinions on deepening the reform of the healthcare security system in order to comprehensively establish a healthcare security system with Chinese characteristics, and endeavor to solve the problem of imbalance and insufficiency in the development of medical security. Healthcare insurance is a major institutional arrangement that reduces the burden of medical treatment on the public, enhances people’s well-being, and maintains social harmony and stability; by 2030, China will have fully established a medical insurance system with basic medical insurance as the mainstay, medical assistance as the backbone, and supplemental medical insurance, commercial health insurance, charitable donations, and medical mutual aid as co-development.

### 3.2 Spatial pattern of healthcare resource allocation

Using AHP and comprehensive score method to measure the comprehensive score of healthcare resource allocation and the score of secondary indicators of 31 provincial administrative units across the country in 2010, 2015, 2018 and 2021, and visualize them through GIS to reveal their spatial–temporal pattern and spatial differentiation law.

**Figure 3:**
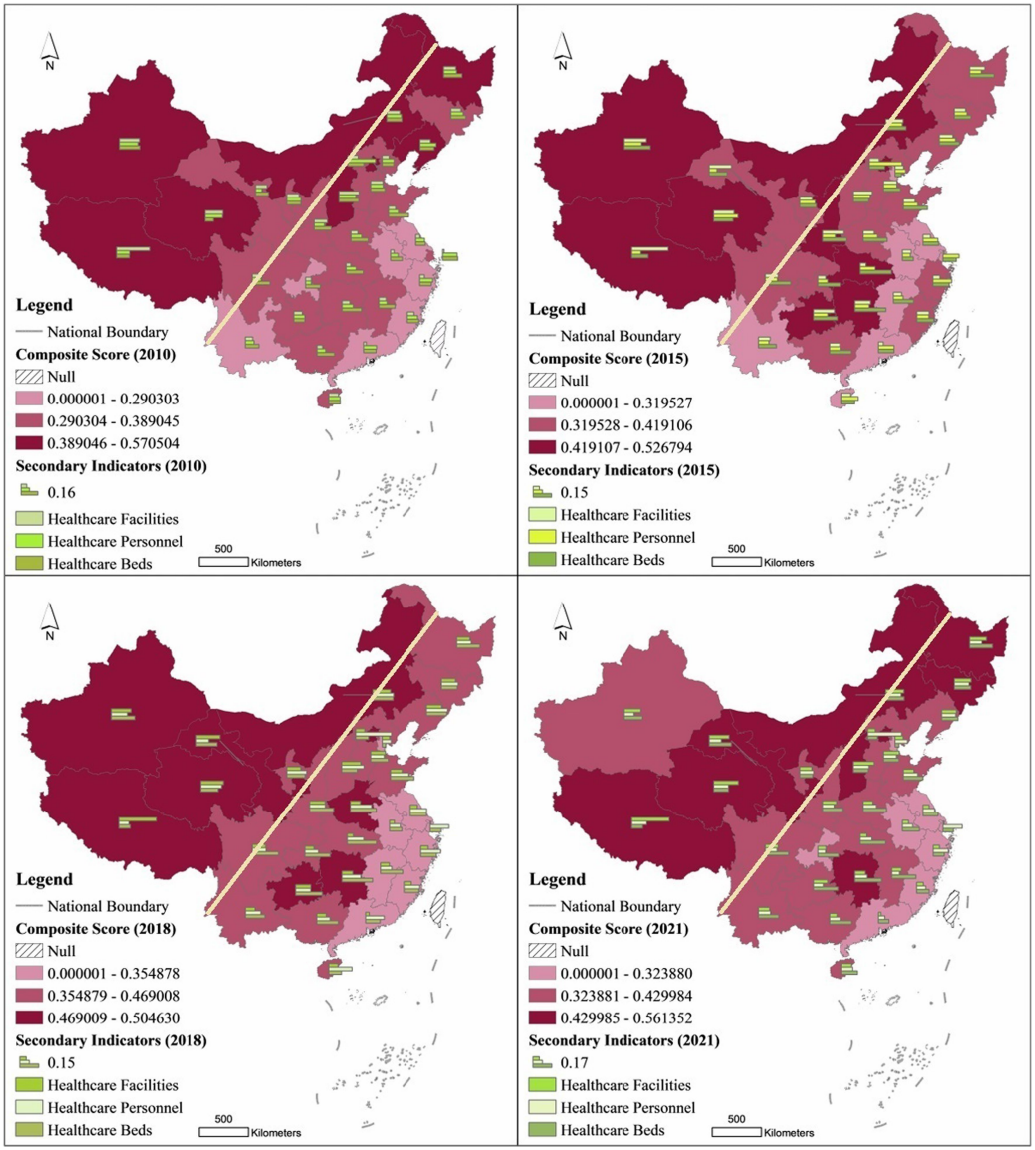
Spatial pattern of healthcare allocation scores by province in China.

First, the spatial pattern of healthcare resource allocation composite scores by province was analyzed. The study found that the healthcare resource allocation composite scores of Chinese provinces are distributed along both sides of the Hu Huanyong line (the Heihe-Tengchong line, east of which more than 90% of China’s population is concentrated, is the Hu Huanyong line). Provinces to the left of the Hu Huanyong line generally have higher healthcare resource allocation composite scores, while provinces to the right of the Hu Huanyong line have lower healthcare resource allocation composite scores (Figure 3). Among the top 25% of provinces ranked by the composite score, the Northeast region slipped from 2 to 1 province, the Eastern region had only 1 provincial administrative unit, the Central region rose from 1 to 2 provinces, and the Western region experienced a rise from 4 to 6 provinces and then slipped to 4 provinces. Among the bottom 25% of provinces in terms of overall score, the eastern region rises from 5 to 6 provinces, the central region varies between 1 and 2 provinces, and the western region declines from 2 to 1. In 2010, the top 5 provinces were Tibet, Xinjiang, Shanxi, Beijing, and Heilongjiang, and the bottom 5 provinces were Jiangsu, Anhui, Chongqing, Guangdong, and Fujian, in that order. 2021, the top 5 provinces are Tibet, Qinghai, and Fujian, in that order. In 2021, the top 5 provinces are Tibet, Qinghai, Gansu, Inner Mongolia and Heilongjiang, and the bottom 5 provinces are Guangdong, Tianjin, Fujian, Shanghai and Zhejiang (Table 3).

**Table 3:** Ranking of healthcare resource allocation composite scores by province in China.

Year	Sorting	Northeastern Region	Eastern Region	Central Region	Western Region
2010	Top25%	2	1	1	4
	Middle50%	1	4	4	6
	Bottom25%	0	5	1	2
2015	Top25%	0	1	1	6
	Middle50%	3	4	3	5
	Bottom25%	0	5	2	1
2018	Top25%	0	1	1	6
	Middle50%	3	3	3	6
	Bottom25%	0	6	2	0
2021	Top25%	1	1	2	4
	Middle50%	2	3	3	7
	Bottom25%	0	6	1	1

Second, the spatial pattern of healthcare resource allocation secondary indicator scores by province is analyzed. In terms of Healthcare Personnel’s secondary indicator scores, the top 5 provinces in 2010 were Beijing, Xinjiang, Qinghai, Shanxi, and Liaoning, and the bottom 5 provinces were Chongqing, Gansu, Anhui, Jiangxi, and Guangxi, in that order. In 2021, the top 5 provinces were Beijing, Shanghai, Shanxi, Inner Mongolia, and Zhejiang, and the bottom 5 provinces were Guangdong, Chongqing, Jiangxi, Fujian, and Guizhou, in that order. In terms of the Healthcare Facilities secondary indicator scores, the top 5 provinces in 2010 were Tibet, Shanxi, Xinjiang, Qinghai, and Inner Mongolia, and the bottom 5 provinces were Shanghai, Guangdong, Anhui, Jiangsu, and Hubei, in that order. The top 5 provinces in 2021 were Tibet, Qinghai, Shanxi, Gansu, and Inner Mongolia, and the bottom 5 provinces were Zhejiang, in that order, Jiangsu, Anhui, Guangdong and Shanghai. In terms of the Healthcare Beds secondary indicator scores, the top five provinces in 2010 were Xinjiang, Shandong, Shanxi, Hunan, and Heilongjiang, and the bottom five provinces were Fujian, Hainan, Guizhou, Jiangsu, and Qinghai, in that order. In 2021, the top five provinces were Hunan, Hubei, Guizhou, Jiangxi, and Sichuan, and the bottom five provinces were Tibet, Guangdong, Shanghai, Beijing, and Tianjin, in that order. The next five provinces were Tibet, Guangdong, Shanghai, Beijing, and Tianjin.

Characterizations of the evolution of the spatial pattern of healthcare resource allocation in each province:

First, the number of hospitals and the number of hospital beds increased significantly. The number of hospitals in the northeastern, eastern, central and western regions increased from 2,306, 7,303, 4,982 and 6,327 in 2010 to 3,456, 12,808, 9,042 and 11,072 in 2021, respectively. The number of hospitals per 10,000 people in the Northeast, East, Central and West regions increased from 0.211, 0.165, 0.153 and 0.206 in 2010 to 0.356, 0.240, 0.259 and 0.321, respectively, in 2021, beds in primary healthcare institutions were 9.471, 7.737, 15.041 and 12.967, respectively.

Second, the comprehensive score of healthcare resource allocation shows a spatial pattern of “west high, east low.” The reason for the “west-high-east-low” composite score by province is closely related to population density. Our study focuses on reflecting the level of healthcare resource allocation between regions, and uses the average volume indicator to measure the total resources of hospital facilities in the eastern region may not be inferior to those in the western region, but the western region is sparsely populated and the eastern region has a high population density, which leads to the spatial phenomenon of “west high, east low” in the average volume indicator of the two regions. In 2021, the population density of Tibet Autonomous Region, which ranked first in terms of Healthcare Facilities secondary indicator score, was 2.5 people per square meter, while the population density of Shanghai, which ranked last in terms of Healthcare Facilities secondary indicator score, was 3,900 people per square meter, which is a difference of 1,560 times. In 2010, the number of hospitals per 10,000 people in Tibet was 0.337, and the number of hospitals per 10,000 people in Shanghai was 0.133, with a difference of 2.53 times, while in 2020 the number of hospitals per 10,000 people in the former was 0.489, and the number of hospitals per 10,000 people in the latter was 0.171, and the gap between the two widened to 2.86 times. In 2010, the number of professional public health institutions per 10,000 people in Tibet and Shanghai was 1.140 and 0.592 respectively, and in 2020, the number of beds in professional public health institutions per 10,000 people in Tibet and Shanghai changed to 1.366 and 0.522, respectively. The former’s *per capita* amount of healthcare was expanding, while the latter’s *per capita* amount of healthcare is growing slowly or even declining, due to the high population density in developed eastern regions such as Shanghai, and the size of the population greatly dilutes healthcare resources in developed regions.

Third, the allocation of high-quality healthcare resources presents a spatial pattern of “high in the east and low in the west.” The western region scores higher in healthcare facilities but lower in doctor resources. The opposite is true for the eastern region, which scores lower in terms of hospitals *per capita* and hospital beds *per capita*, but higher in terms of physician resource allocation. 2021 Healthcare Personnel rankings for secondary indicators show that cities such as Beijing and Shanghai are in the top two, while regions such as Chongqing and Xinjiang are at the bottom of the rankings. In addition to the impact of population density on the indicator of the number of doctors per 10,000 people, population migration factors are also playing a huge role. Regions such as Beijing and Shanghai are national center cities with the most advanced healthcare resources in China, bringing together the best doctors in the country. In fact, the concentration of healthcare professionals and technicians is closely related to economic development, with talents tending to concentrate in more developed regions, which also provide healthcare professionals and technicians with considerable salaries, a platform to help the world and the people, and a platform to show their skills, which is in line with the law of migration of talents.

### 3.3 Spatial and temporal analysis of healthcare resource allocation

Using spatial autocorrelation analysis to test the distribution of high and low concentrations of healthcare resource allocation, we calculated and tested the global Moran’s I statistic for the composite scores of healthcare resource allocation in 31 provincial administrative units in China. As shown in Table 4, the global Moran’s I statistic of healthcare resource allocation composite score of each province in China from 2010 to 2021 passes the significance test at the 0.01 confidence level, and the global Moran’s I statistic is greater than zero, that’s the healthcare resource allocation composite score of each province in China is in the global distribution of high and low agglomeration. Resource allocation composite scores in China’s provinces have positive spatial correlation at the global level. This suggests that at the national level, provinces with higher healthcare resource allocation composite scores are geographically adjacent to provinces that also have higher healthcare resource allocation composite scores, and provinces with lower healthcare resource allocation composite scores are geographically adjacent to provinces that also have lower healthcare resource allocation composite scores.

**Table 4:** Moran’s I statistics by Province, China, 2010–2021.

Variables	2010	2015	2018	2021
Moran’s I	0.4500**	0.3742**	0.4120**	0.5250**
variance	0.0063	0.0062	0.0062	0.0062
Z-statistic	6.0680	5.1337	5.6293	7.0421

**Moran’s I statistic for each year passed the significance test at the 0. 01 significance level.

The global Moran’s I statistic shows a “decreasing-increasing-increasing” trend over the sample period of 2010–2021. This indicates that, globally, the spatial agglomeration effect of provinces with similar healthcare resource allocation scores shows a development trend of “weakening and then strengthening.” From the specific period, there is a significant decline in 2010–2015, with the global Moran’s I statistic decreasing from 0.4500 to 0.3742, and then a slow increase from 2015 to 2018, with the Moran’s I statistic increasing sharply from 2018–2021 to 0.4120. 0.4120 to 0.5250 sharply.

**Figure 4:**
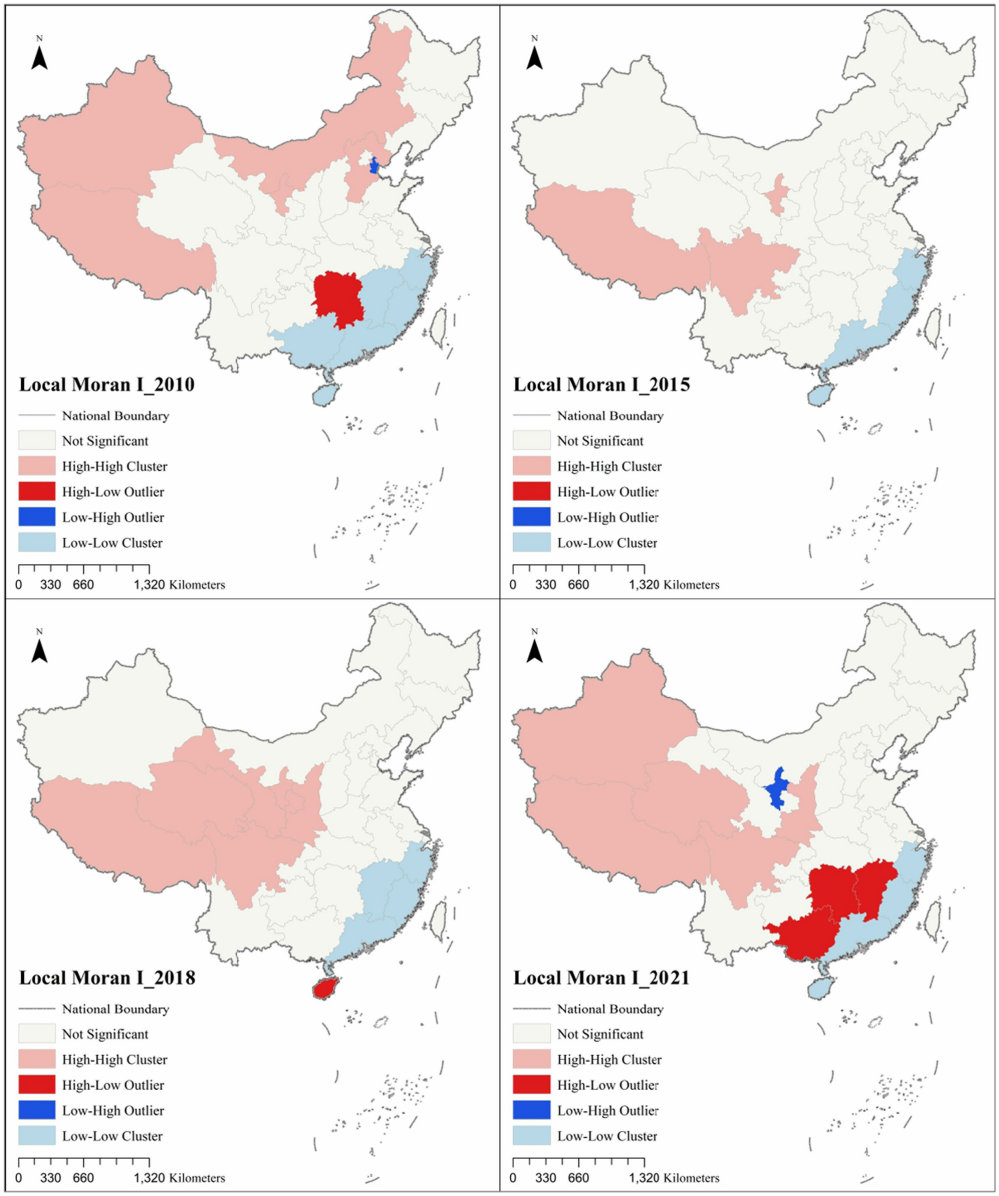
Healthcare resource allocation LISA cluster by province, 2000–2021.

In order to deeply explore the spatial agglomeration of the composite score of healthcare resource allocation and its spatial–temporal evolution in each province of China, further spatial statistical analyzes of the composite score of healthcare resource allocation in local areas were conducted. Using the local spatial autocorrelation technique, healthcare resource allocation in China was analyzed, and the LISA clustering map of the composite score of healthcare resource allocation was plotted (Figure 4; Table 5).

**Table 5:** Healthcare resource allocation cluster type by province, 2010–2021.

Year	HH cluster	HL cluster	LH cluster	LL cluster
2010	Xinjiang, Tibet, Inner Mongolia, Ningxia, Hebei	Hunan	Tianjin	Zhejiang, Fujian, Jiangxi, Guangdong, Guangxi, Hainan
2015	Tibet, Sichuan, Ningxia			Zhejiang, Fujian, Guangdong, Hainan
2018	Tibet, Qinghai, Gansu, Ningxia, Shaanxi, Sichuan	Hainan		Zhejiang, Fujian, Guangdong, Jiangxi
2021	Xinjiang, Tibet, Qinghai, Sichuan, Shaanxi	Jiangxi, Hunan, Guangxi	Ningxia	Zhejiang, Fujian, Guangdong, Hainan

From the four time-image LISA cluster maps of 2010–2021, HH cluster and LL cluster have obvious pointing characteristics of Hu Huanyong line, the left side of Hu Huanyong line mainly involves 7 western provinces such as Xinjiang, Tibet, Qinghai, Inner Mongolia, Gansu, Ningxia, Sichuan, etc., and the right side of Hu Huanyong line mainly involves 24 regional provinces such as Beijing, Shanghai, Guangdong, Hunan, Hubei, Guangxi, etc. In 2010, there are 13 provinces with significant local spatial autocorrelation analysis, of which 6, 2 and 5 are in the eastern region, central region and western region, respectively. In 2010, the number of provinces with significant local spatial autocorrelation analyzes was 13, of which 6, 2 and 5 were in the eastern, central and western regions, respectively. The provinces in the HH cluster were Xinjiang, Tibet, Inner Mongolia, Ningxia and Hebei, mainly concentrated on the left side of Hu Huanyong line, while the provinces in the LL cluster were Zhejiang, Fujian, Jiangxi, Guangdong, Guangxi and Hainan, all of which were concentrated on the left side of Hu Huanyong line. Guangxi, and Hainan, all of which are concentrated on the right side of the Hu Huanyong line, in addition to Hunan for the HL cluster, and Tianjin for the LH cluster. In 2015, seven provinces passed significant local spatial autocorrelation analyzes, of which four and three were in the eastern and western regions, respectively. The provinces in the HH cluster were Tibet, Sichuan, and Ningxia, all of which were concentrated on the left side of Hu Huanyong line, whereas the provinces in the LL cluster were Zhejiang, Fujian, Guangdong, and Hainan, all of which were concentrated on the right side of Hu Huanyong line. In 2018, the number of provinces whose local spatial autocorrelation analyzes passed as significant was 11, of which the eastern region, the central region, and the western region accounted for 4, 1, and 6, respectively. The provinces in the HH cluster are Tibet, Qinghai, Gansu, Ningxia, and Shaanxi, Sichuan, mainly concentrated on the left side of the Hu Huanyong line, while the provinces in the LL cluster are Zhejiang, Fujian, Guangdong, and Jiangxi, all of which are concentrated on the right side of the Hu Huanyong line, in addition to the province in the HL cluster, Hainan. In 2021, the local spatial autocorrelation analyzes pass the significant provinces, of which the eastern, central, and western regions accounted for 4, 2, and 7, respectively. The provinces in the HH clusters are Xinjiang, Tibet, Qinghai, Sichuan, and Shaanxi, which are mainly concentrated on the left side of the Hu Huanyong line, while the LL clusters are Zhejiang, Fujian, Guangdong, and Hainan, all of which are concentrated on the right side of the Hu Huanyong line, in addition to the HL clusters of Jiangxi, Hunan, and Guangxi, and the LH cluster of provinces is Ningxia.

Overall, the provinces with spatial autocorrelation and passing the significance test undergo changes in spatial patterns over time. The HH cluster provinces that passed the significance test were mainly distributed on the left side of the Hu Huanyong line, indicating that the healthcare resource allocation in the provinces on the left side of the Hu Huanyong line, such as Tibet, Xinjiang, Qinghai, Ningxia, Gansu, Inner Mongolia, Sichuan, geographically had the spatial characteristics of the HH cluster, whereas the LL cluster provinces that passed the significance test were mainly distributed on the right side of the Hu Huanyong line, indicating that the healthcare resource allocation in the southeastern coastal provinces, such as Zhejiang, Fujian, Guangdong, Hainan, geographically had the spatial characteristics of the LL cluster. The local spatial autocorrelation analysis of the healthcare resource allocation composite scores was verified with the above spatial pattern of “high in the west and low in the east” in the healthcare resource allocation composite scores, to reveal the spatial and temporal variations in China’s healthcare resource allocation, and to provide empirical evidence for the optimal allocation of China’s healthcare resources.

### 3.4 Quadrant analysis of healthcare resource allocation

Considering the dynamic changes in healthcare resource allocation, we adopt a two-dimensional quadrant analysis method, dividing the healthcare resource allocation of 31 provinces into four quadrants from 2010 to 2021, to reveal the dynamic characteristics of healthcare resource allocation in each province. The origin of the coordinates in the four quadrants is the average of the composite scores of healthcare resource allocation in 2010 and 2021. Quadrant I indicates that healthcare resourcing in a province is above average in both 2010 and 2021 time points; Quadrant II indicates that healthcare resourcing in a province is below average in 2010 but above average in 2021; Quadrant III indicates that healthcare resourcing in a province is below average in both 2010 and 2021 time points; and Quadrant IV indicates that healthcare resourcing in a province is above average in 2010 but below average in 2021.

**Figure 5:**
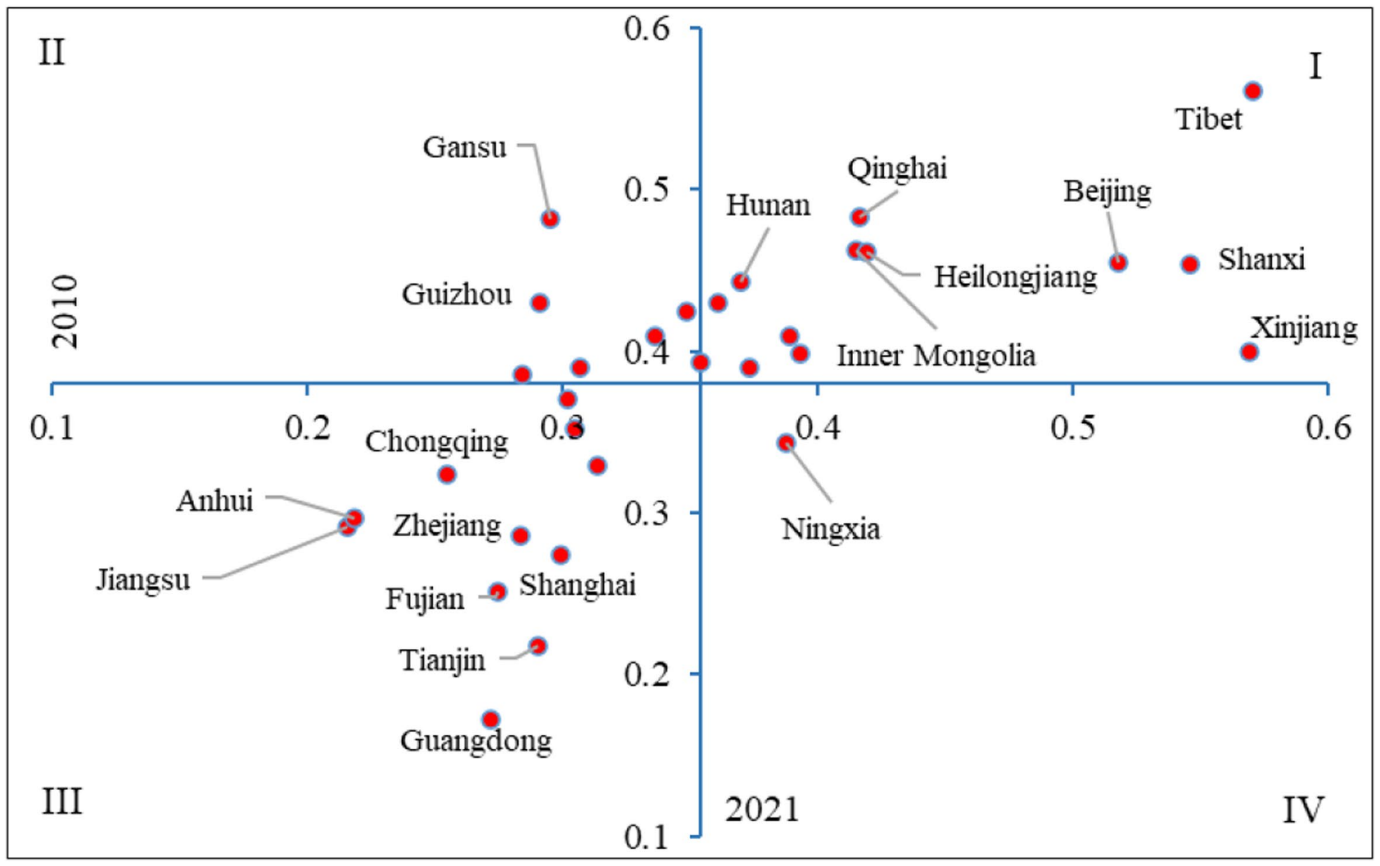
Two-dimensional quadrant classification of healthcare allocation composite scores by province in China.

The spatial differentiation of the composite score of healthcare resource allocation in China’s 31 provinces in 2010–2021 is obvious (Figure 5), which is manifested in three aspects: firstly, the number of provincial units that are higher or lower than the mean of the composite score of healthcare resource allocation in the two-time nodes of 2010–2021 are 13 (located in the first quadrant) and 11 (located in the third quadrant), respectively, which shows a polarization trend. Secondly, the number of provinces with improved healthcare resourcing composite scores is higher than the number of provinces with declining composite scores, that’s provincial cells located in Quadrant II (6) are higher than provincial cells in Quadrant IV (1). Thirdly, the largest number of provinces in the western region (5) are located in the first quadrant, and the largest number of provinces in the eastern region (7) are located in the third quadrant, while the fact that three provincial units in the eastern region, including Beijing, Hebei, and Shandong, are located in the first quadrant, is an important reason for the large variations within the eastern region.

Healthcare resource allocation is located in the first quadrant I for 13 provinces, including 3 provinces in the northeastern region, 3 provinces in the eastern region, including Beijing, Hebei, and Shandong, 5 provinces in the west region, including Xinjiang, Tibet, Inner Mongolia, Shaanxi, and Qinghai, and 2 provinces in the central region, including Shanxi and Hunan. Healthcare resource allocation is located in Quadrant II for 6 provinces, including two provinces in the central region, that’s Henan and Hubei, and four provinces in the western region, that’s Sichuan, Guizhou, Yunnan, and Gansu. Healthcare resource allocation is located in Quadrant III for 11 provinces, including 7 provinces in the eastern region (Tianjin, Shanghai, Jiangsu, Zhejiang, Fujian, Guangdong, Hainan, etc.), 2 provinces in the western region (Guangxi and Chongqing), and 2 provinces in the central region (Anhui and Jiangxi). Healthcare resource allocation is located in the fourth quadrant (IV), which is located in the western region, but only in the province of Ningxia province.

Further analysis of healthcare resource allocation in the four quadrant provinces:

Quadrant 1 case province Tibet. As a western province with a low population density, Tibet has the highest composite score for healthcare resource allocation in the country. Between 2010 and 2021, Tibet’s Healthcare Facilities score increased from 0.3200 to 0.3324, the Healthcare Personnel score increased from 0.1261 to 0.1324, and the Healthcare Beds score decreases from 0.1244 to 0.0965. Although the comprehensive score of Tibet’s healthcare resource allocation decreases from 0.5710 in 2010 to 0.5610 in 2021, it still maintains the first place in the country, indicating that Tibet is better developed in terms of the quantity of healthcare resource allocation. Of course, Tibet also has shortcomings, such as a low Healthcare Beds score, indicating that there is still room for further improvement in the quality of its healthcare resource allocation.

Quadrant 2 case province Gansu. As a western province with a low population density, Gansu’s healthcare resource allocation composite score rises rapidly from 22nd in 2010 to 3rd in 2021.Between 2010 and 2021, Gansu’s Healthcare Facilities score grows from 0.1102 to 0.1770, and Healthcare Personnel score grows from 0.0606 to 0.1075. The highest contribution to Gansu’s composite score is made by the Healthcare Beds score, which increases from 0.1247 to 0.1971. From 2010 to 2021, Gansu’s Healthcare Facilities score increases from 0.1102 to 0.1770, Healthcare Personnel score from 0.0606 to 0.1075, and Healthcare Beds score from 0.1247 to 0.1971. The highest contribution to Gansu’s overall score is made by the Healthcare Beds score, reflecting that Gansu has been comprehensively improved in healthcare resource allocation.

Quadrant 3 case province Guangdong. As an eastern province with a high population density, Guangdong’s healthcare resource allocation composite score ranks rather poorly, as it drops from 28th in 2010 to 31st in 2021.Between 2010 and 2021, Guangdong’s Healthcare Facilities score decreases from 0.0259 to 0.0224, and its Healthcare Facilities score of Guangdong decreases from 0.1251 to 0.0566, Healthcare Personnel score from 0.1207 to 0.0934. The decrease of Healthcare Facilities score of Guangdong is not large, but the decrease of Healthcare Personnel and Healthcare Beds scores is large, reflecting that the *per capita* healthcare in Guangdong is not as large as it should be. The decrease in Healthcare Facilities score is not large, but the decrease in Healthcare Personnel and Healthcare Beds scores is large, reflecting the low *per capita* healthcare resources in Guangdong, which to a certain extent reveals a large contradiction between people’s demand for healthcare resources and the supply of healthcare resources in Guangdong.

Quadrant 4 case province Ningxia. As a province in the western region, Ningxia has a low population density, but its healthcare resource allocation composite score ranking drops from 10th in 2010 to 25th in 2021.Between 2010 and 2021, Ningxia’s Healthcare Facilities score decreases from 0.1167 to 0.1063, Healthcare Personnel score decreases from 0.1369 to 0.1226, and Healthcare Beds score decreases from 0.1340 to 0.1140.Ningxia’s Healthcare Facilities, Healthcare Personnel, and Healthcare Beds scores all decrease, resulting in a decline in the overall score ranking at a significant rate, reflecting Ningxia’s decline in recent years. The speed is obvious, reflecting the fact that Ningxia’s healthcare resources in recent years have been developing at a slower pace than the national average, both in terms of quantity and quality.

The first quadrant, in which the allocation of health care resources in a province is above average in both 2010 and 2021, concentrates most western provinces, while the third quadrant, in which the allocation of health care resources in a province is below average in both 2010 and 2021, concentrates most eastern provinces, reflecting spatial and temporal variations in the allocation of health care resources in the four major zones. From the perspective of balanced development, the four major zones tend to be in a relatively balanced state in terms of the quantity of healthcare resource allocation, but there are still gaps in the quality of regional healthcare resource allocation. The eastern region is still the region where high-quality health-care resource allocation is concentrated, while the western region still has room for further improvement in the quality of its health-care resource allocation for historical, economic and other reasons.

## 4 Discussion

### 4.1 Quantitative and qualitative balance in the allocation of healthcare resources

The allocation of healthcare resources in China’s four major zones has undergone a process of change from “unbalanced quantity to relatively balanced quantity,” with a spatial pattern of “high in the west and low in the east” in terms of the composite score of healthcare resources, and a spatial pattern of “high in the east and low in the west” in terms of the high quality of healthcare resources. According to Fudan University’s 2021 National Top 100 Hospitals Ranking, China’s leading tertiary hospitals are highly concentrated in Beijing (22), Shanghai (19), Guangzhou (10), Hangzhou (5), Wuhan (5), Chengdu (4), Nanjing (4), Xi’an (4), Chongqing (4), Tianjin (3), Changsha (3), Fuzhou (2), Harbin (2), Hefei (2), and Jinan (2), Shenyang (2), Zhengzhou (2), Nanchang (1), Qingdao (1), Shenzhen (1), Suzhou (1), Changchun (1) and other provincial capitals and municipalities, which are 22 cities that concentrate the nation’s best healthcare resources. By region, the top 100 hospitals in the country account for 70% in the Central and Eastern regions, 13% in the Central region, 12% in the Western region and 5% in the Northeast. High-quality healthcare resources are highly concentrated in the eastern region, which is consistent with the findings of related scholars. Zhao et al. pointed out that China’s provincial and prefectural-level municipal-scale high-quality healthcare resources are mostly concentrated in the area east of the Hu Huanyong line (22), reflecting that China’s provincial healthcare resource allocation has basically achieved quantitative parity in the development pattern, but qualitatively, it has not achieved parity. This reflects that China has basically achieved equalization in the allocation of healthcare resources by province in terms of quantity, but the spatial pattern of “high in the east and low in the west” in terms of quality is still difficult to change.

### 4.2 Internet plus healthcare resource allocation in the age of artificial intelligence

The high concentration of quality healthcare resources in megacities has its own regularity and inevitability. Such cities are able to provide sufficient financial support for the research and development of healthcare technology, have strong key healthcare disciplines and teams of specialists, and facilitate the technological research of difficult and complicated diseases, which, to a certain extent, contributes to the advancement of healthcare technology across the country and even in the world. However, the high concentration of high-quality healthcare resources has also brought confusion to patients. Famous hospitals in mega cities such as Beijing and Shanghai undertake the rescue and treatment of difficult and complicated diseases in the whole country, resulting in overcrowding in tertiary hospitals in general, and the limited high-quality healthcare resources have been squeezed, and the problem of conflicts in access to healthcare care is particularly prominent. The Opinions on Deepening the Reform of the Medical Security System provides institutional guarantee for solving the problem of unbalanced and inadequate development of healthcare security, while the development of “Internet+” intelligent healthcare in the era of artificial intelligence provides technical means for solving the contradiction between limited high-quality healthcare resources and difficulties in accessing healthcare care. The organic combination of artificial intelligence and the Internet can make the allocation of healthcare resources more efficient, accurate and personalized, and through 5G communication technology, artificial intelligence, Internet of Things technology and cloud platforms, it can carry out remote healthcare diagnosis, remote surgery, emergency guidance, share high-quality healthcare resources and promote the optimal allocation of high-quality healthcare resources.

## 5 Conclusion

The article collects panel data on healthcare resource allocation by province in China from 2010 to 2021, and comprehensively uses the Analytic Hierarchy Process, the composite score method, the regional difference analysis method and the spatial autocorrelation analysis to reveal the regional differences, spatial and temporal patterns and development characteristics of healthcare resource allocation in 31 provincial administrative units in China. The study reveals the regional differences, spatial and temporal patterns and development characteristics of healthcare resource allocation in 31 provincial administrative units in China. The main findings are as follows:

First, in terms of regional differences, the overall scores of healthcare resource allocation in the four major zones show a fluctuating trend of growth, which is manifested in the process of “rising-declining-rising-declining,” with the intra-zone differences in healthcare resource allocation tending to narrow and the inter-zone differences tending to widen. The contribution rate of intra-zone differences declined from 90.93 per cent in 2010 to 62.99 per cent in 2021, while the contribution rate of inter-zone differences increased from 9.07 per cent in 2010 to 37.01 per cent in 2021.

Second, from the perspective of spatial pattern, the high or low scores of healthcare resource allocation in each province in China are bounded by the Hu Huanyong line, and the provinces on the left side of the Hu Huanyong line generally have high scores and show the spatial characteristics of centralized and continuous distribution, while the provinces on the right side of the Hu Huanyong line have relatively low scores and show the spatial characteristics of discrete distribution. The spatial pattern of healthcare resource allocation in each province is characterized by a significant growth rate in the number of hospitals and the number of hospital beds, a spatial pattern of “high in the west and low in the east” in terms of the overall score, and a spatial pattern of “high in the east and low in the west” in terms of high-quality healthcare resources.

Third, from the perspective of spatial and temporal analyzes, the sample period of 2010–2021 shows the development trend of “decreasing-rising-increasing.” From a global perspective, the spatial agglomeration effect of provinces with close scores in healthcare resource allocation shows a development trend of “weakening and then strengthening.” The HH cluster provinces that passed the significance test were mainly distributed on the left side of the Hu Huanyong line, indicating that the healthcare resource allocation in the provinces on the left side of the Hu Huanyong line, such as Tibet, Xinjiang, Qinghai, Ningxia, Gansu, Inner Mongolia, Sichuan, geographically had the spatial characteristics of the HH cluster, whereas the LL cluster provinces that passed the significance test were mainly distributed on the right side of the Hu Huanyong line, indicating that the healthcare resource allocation in the southeastern coastal provinces, such as Zhejiang, Fujian, Guangdong, Hainan, geographically had the spatial characteristics of the LL cluster.

Fourth, from the quadrant analysis, the comprehensive score of healthcare resource allocation in each province is divided into four quadrants, the first quadrant where the healthcare resource allocation in a province is above the average in both 2010–2021-time nodes concentrate most of the western provinces, while the third quadrant, where healthcare resource allocation is below average in both 2010 and 2021, concentrates most of the eastern provinces.

## Data availability statement

Publicly available datasets were analyzed in this study. This data can be found at: http://www.stats.gov.cn/sj/ndsj.

## Author contributions

HR: Conceptualization, Writing – original draft. CL: Writing – review & editing, data curation. YH: Methodology.
